# Immediate hypersensitivity to polyethylene glycols in unrelated products: when standardization in the nomenclature of the components of drugs, cosmetics, and food becomes necessary

**DOI:** 10.1186/s13223-019-0327-4

**Published:** 2019-02-19

**Authors:** Vicente Jover Cerdá, Ramón Rodríguez Pacheco, Joan Doménech Witek, Francisco Manuel Marco de la Calle, María Luz de la Sen Fernández

**Affiliations:** 1 Sección de Alergología, Hospital General Universitario de Elda, Ctra. De Sax, s/n, 03600 Elda, Alicante Spain; 2 Servicio de Análisis Clínicos e Inmunología, Hospital General Universitario de Alicante, Alicante, Spain

**Keywords:** Excipient allergy, Anaphylaxis, Basophil activation test, Hypersensitivity, Polyethylene glycol

## Abstract

**Background:**

Polyethylene glycols (PEGs) and their derivatives are non-ionic polymers of ethylene oxide commercially available with numerous synonyms, such as macrogol, oxyethylene polymer, and laureth-9. Although these polymers are usually safe, mild to life-threatening immediate-type hypersensitivity reactions have been reported. Nevertheless, awareness about their allergic potential is minimal due to the non-standardization of their nomenclature.

**Case presentation:**

We present the case of a 29-years-old woman who developed several local and systemic type I hypersensitivity reactions including a severe anaphylactic reaction to different pharmacologic and cosmetic products whose excipients included PEG. Prick tests and basophil activation tests were performed to several pharmacological and cosmetic products, but only those containing PEGs and their derivatives were positive. The patient was diagnosed with immediate hypersensitivity IgE-mediated to PEGs and its derivatives.

**Conclusions:**

Standardization of the terminology used to describe the presence of PEGs in products would help patients to identify them clearly and unequivocally and thus avoid the development of hypersensitivity reactions. It is also recommended studying PEG allergy in reactions to products containing PEGs, once allergy to the active ingredients has been excluded and in reactions to multiple unrelated drugs.

*Clinical study protocol number* PI2018/29 (registered on 24 September 2018)

## Background

Polyethylene glycols (PEGs) and their derivatives are non-ionic polymers of ethylene oxide commercially available over a wide range of molecular weights from 200 g/mol to 35,000 g/mol and widely used in medical, pharmaceutical, cosmetic, industrial, and food products [1, 2]. These polymers have numerous synonyms, such as macrogol, oxyethylene polymer, and laureth-9 [2]. Nevertheless, the term “PEG” is often used in combination with a number referring to the number of ethylene oxide units (cosmetic industry) or to the molecular weight (pharmaceutical industry) [2].

Since its development, PEG polymers held a reputation for safety, nevertheless from mild to life-threatening immediate-type hypersensitivity reactions have been reported, with clinical manifestations ranging from generalized urticaria to anaphylactic shock [2–5]. Awareness about the allergenic potential of these polymers is minimal due to the non-standardization of their nomenclature, inadequate labelling of products containing PEGs, and the lack of suspicion as the agents responsible of such reactions. In fact, no studies have examined the prevalence of type 1 PEGs hypersensitivity, so its incidence may have been underestimated.

## Case presentation

We present the case of a 29-years-old woman with history of atopic eczema and contact dermatitis by nickel sulfate, subclinical sensitization to mites and cypress, and cholinergic urticaria. She developed several local and systemic type I hypersensitivity reactions including a severe anaphylactic reaction to different pharmacologic and cosmetic products whose excipients included PEGs.

Two years before consultation, the patient developed generalized urticaria, dizziness, and dyspnea 30 min after using a skin antiseptic (Betadine^®^ solution: iodopovidone and laureth-9 as excipient). Symptoms improved after treatment with dexchlorpheniramine and methylprednisolone. Six months later, 30 min after swallowing 30 ml of a cough syrup (GripaNait^®^: paracetamol, dextromethorphan, and doxylamine as active ingredients and several excipients, including macrogol 6000), she developed generalized pruritus, dyspnea, severe dizziness, seizures, loss of consciousness, and respiratory arrest, requiring urgent treatment with adrenaline, plasma expanders, and parenteral corticosteroids. In the last 7 years she developed itchy maculopapular rashes in contact with some moisturizing skin creams containing PEG-75 and PEG-100. In May 2017, she reported generalized urticaria after applying soap to a tattooed area and wheals after applying a moisturizing creams on intact skin. In November 2017, she experienced swelling of the gums and tongue after using a toothpaste for which she did not need treatment.

An allergological study was carried out with her prior consent. Levels of C3, C4, IgA, IgG, IgM, and tryptase were all within normal range. Prick test and specific IgE were positive for mites and cypress, but negative for other aeroallergens, latex, anisakis, and several foods. Specific IgE was also negative for ethylene oxide. We detected 1626 IU/ml of total IgE.

**Fig. 1:**
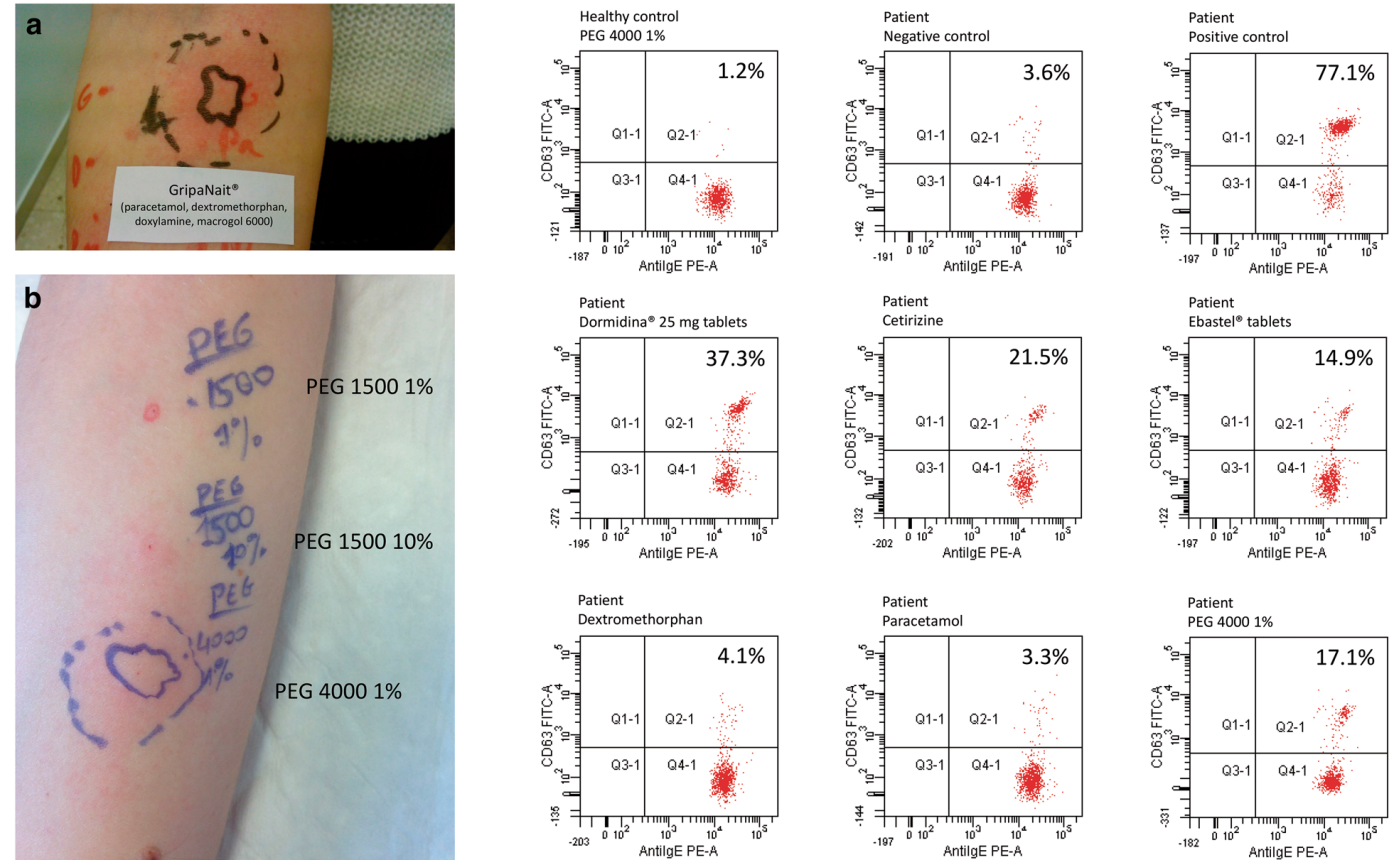
Skin prick test and basophil activation test results. Skin prick test (left): **a** cough syrup GripaNait^®^, containing paracetamol, dextromethorphan, doxylamine, and macrogol 6000; **b** PEG 1500 1% and 10% (negative), and PEG 4000 1% (positive). Basophil activation test results (right): Q2-1 represents activated basophils CD63+IgE+ (% indicated) and Q4-1 represents non-activated basophils CD63−IgE+. Positive control used anti-IgE antibody and negative control used isotonic solution

Prick tests with GripaNait^®^ (Fig. 1a) and Betadine^®^ gel and solution were positive. Prick tests with each of their ingredients separately were negative, but positive for PEGs and doxylamine. To test doxylamine separately, we used Dormidina^®^ 25 mg. Doxylamine is a histamine H1 receptor antagonist belonging to the ethanolamines group, such as diphenhydramine. To date few cases of allergy to these antihistamines have been described. One of such cases developed anaphylaxis to diphenhydramine included in the intranasal drops Coldistan^®^ [6]. It is important to remark that Coldistan^®^ contains PEG as excipient.

We noticed that the preparation of doxylamine used in our prick test (Dormidina^®^ 25 mg) contained PEG 8000. For this reason, we retested doxylamine prick test with Cariban^®^ tablets, a drug used as antiemetic that contains doxylamine plus pyridoxine, but without any PEG. Prick test with this drug resulted negative, as well as other antihistamines without PEG such as chlorpheniramine, diphenhydramine tablets, hydroxyzine, mepyramine, cetirizine solution, bilastine, and loratadine. Table 1 shows other products tested in our study. Of note, only products containing PEGs resulted positive in the prick tests. We also performed a basophil activation test (BAT) to the products listed in Table 1. Only products containing PEGs resulted positive, except the toothpaste containing PEG-6 that was negative, probably due to cytotoxic effects. All tests were compared with healthy volunteers resulting negative in all of them (Fig. 1). Negativity of BAT due to toxic effects was controlled by assaying a battery of decreasing concentrations of antigen. Non-specific activation was ruled out by testing healthy volunteers.

**Table 1:** Products assessed by skin prick test and basophil activation test

Drugs (active ingredients)	Contains PEG	Prick test	BAT
GripaNait^®^ cough syrup (paracetamol, dextromethorphan, doxylamine)	+ (macrogol 6000)	+	NP
Betadine^®^ solution (iodopovidone)	+ (laureth-9)	+	NP
Betadine^®^ gel (iodopovidone)	+ (PEG 400, 4000 and 6000)	+	NP
Romilar^®^ tablets (dextromethorphan)	−	−	−
Dormidina^®^ 25 mg tablets (doxylamine)	+ (PEG 8000)	+	+
Dormidina^®^ 12.5 mg tablets (doxylamine)	+ (PEG 400 and 6000)	Weak +	NP
Cariban^®^ tablets (doxylamine, pyridoxine)	−	−	NP
Polaramine^®^ solution (dexchlorpheniramine)	−	−	−
Soñodor^®^ tablets (diphenhydramine)	−	−	−
Atarax^®^ solution (hydroxyzine)	−	−	NP
FLUIDASA^®^ solution (mepyramine)	−	−	−
Cetirizine solution (cetirizine)	−	−	NP
CETIRIZINE tablets (cetirizine)	+ (macrogol 4000)	+	+
Ebastel^®^ tablets (ebastine)	+ (macrogol 6000)	+	+
Ebastel^®^ solution (ebastine)	+ (oxyethylene polymer)	+	+
Bilaxten^®^ tablets (bilastine)	−	−	−
Loratadine tablets (loratadine)	−	−	NP
Movicol^®^ powder (macrogol 3350, sodium chloride, sodium bicarbonate, potassium chloride)	+ (macrogol 3350)	+	+
PEG 1500 (1% and 10%)	+ (PEG 1500)	−	NP
PEG 4000 (1%)	+ (PEG 4000)	+	+
ORAL-B^®^ toothpaste	+ (PEG-6)	−	−
Ziverel^®^ powder (hyaluronic acid, chondroitin sulfate)	+ (poloxamer 407)	+	+
Polysorbate 80 (1% and 20%)	+ (polysorbate 80)	− in 1% + in 20%	− at 0.002 mg/ml + at 0.02 mg/ml
Citrafleet^®^ powder (sodium picosulfate, magnesium oxide, citric acid)	−	−	−
Paracetamol tablets	−	−	−

*BAT* basophil activation test, *NP* not performed, *PEG* polyethylene glycol

As all the products testing positive in the allergy work up contained PEGs (Table 1), its involvement as a causative agent in these reactions was confirmed with pure PEGs of different molecular weights and PEG-derivatives (poloxamer 407 contained in Ziverel^®^ and polysorbate 80) according to the algorithm proposed by Wenande et al. [2] for the investigation of patients with suspected immediate-type PEG hypersensitivity. PEG used was of analytical grade and purchased from Merck (Merck, Darmstadt, Germany). The test was negative with PEG 1500 1% and 10%, but positive with PEG 4000 1% (Fig. 1b). PEG-derivatives also resulted positive in the prick test. BAT resulted positive with PEG 4000 1% and PEG-derivatives (Fig. 1). Controls were done in healthy volunteers resulting negative in all of them. Taking into account that there are studies that have reported delayed hypersensitivity reactions to PEGs [7], a patch test was performed with Betadine^®^ solution, GripaNait^®^, Ziverel^®^, polysorbate 80, and PEG 4000 10%, but it was negative in all products.

The patient was diagnosed with immediate hypersensitivity IgE-mediated to PEG and its derivatives of different molecular weights contained in pharmacological and cosmetic products, with severe anaphylaxis to cough syrup (containing PEG 6000), moderate anaphylaxis to a skin antiseptic (containing PEG-9), contact urticaria or generalized urticaria to moisturizing skin creams (PEG-75 and PEG-100, respectively), contact angioedema by toothpaste (PEG-6), and subclinical skin and in vitro (BAT) sensitivity to poloxamer 407 and polysorbate 80. Interestingly, she does not currently show problems with foods that may contain such products. After recommending avoidance measures to such products by providing her with a list of PEG-free products and their derivatives, she has not experienced any further allergic reactions in the last year. It was recommended to carry on an emergency kit including an auto-injectable adrenaline shot.

## Discussion and conclusion

Although this type of hypersensitivity reactions have been previously described in 37 patients included in the study of Wanande et al. [2] only 4 were assessed through the BAT. In our case we demonstrated an immediate hypersensitivity IgE-mediated to PEG by positive skin prick test and positive BAT. We ruled out delayed hypersensitivity with a negative patch test.

As in 2 of 37 patients of Wanande trial, we didn’t find specific IgE against ethylene oxide [2]. However, our results give limited information on the safety of ethylene oxide for patients sensitized to PEG. IgE test was negative, but we cannot rule out a potential reaction in vivo. Nevertheless, the lesser reactivity observed when assaying PEGs of decreasing molecular weight, may indicate that monomeric ethylene oxide could be devoid of allergenicity by itself, unless conjugated to a complex carrier molecule (i.e. a hapten-carrier mechanism).

The lack of standardization in the nomenclature of PEGs and lack of knowledge about the involvement of PEGs in hypersensitivity reactions means that many patients are not properly diagnosed and develop adverse reactions to many unrelated products. We recommend standardizing the terminology used to describe the presence of PEG in products to avoid confusions and studying PEG allergy in reactions to products containing PEG, once allergy to the active ingredients has been excluded and in reactions to multiple unrelated drugs.

## Abbreviations

BAT: basophil activation test
PEG: polyethylene glycol
